# Transcranial magnetic stimulation reveals two functionally distinct stages of motor cortex involvement during perception of emotional body language

**DOI:** 10.1007/s00429-014-0825-6

**Published:** 2014-07-15

**Authors:** Sara Borgomaneri, Valeria Gazzola, Alessio Avenanti

**Affiliations:** 1 Department of Neuroscience, University Medical Center Groningen and University of Groningen, Groningen, The Netherlands; 2 IRCCS Fondazione Santa Lucia, 00179 Rome, Italy; 3 The Netherlands Institute for Neuroscience, and Institute of the Royal Netherlands Academy of Arts and Sciences (KNAW), Amsterdam, The Netherlands; 4 Centro studi e ricerche in Neuroscienze Cognitive, Dipartimento di Psicologia, “Alma Mater Studiorum” Università di Bologna, Campus di Cesena, Viale Europa 980, 47521 Cesena, Italy

**Keywords:** Motor cortex, Transcranial magnetic stimulation, Motor evoked potentials, Emotion, Body expressions, Action simulation, Embodied cognition, Temporal dynamics

## Abstract

Studies indicate that perceiving emotional body language recruits fronto-parietal regions involved in action execution. However, the nature of such motor activation is unclear. Using transcranial magnetic stimulation (TMS) we provide correlational and causative evidence of two distinct stages of motor cortex engagement during emotion perception. Participants observed pictures of body expressions and categorized them as happy, fearful or neutral while receiving TMS over the left or right motor cortex at 150 and 300 ms after picture onset. In the early phase (150 ms), we observed a reduction of excitability for happy and fearful emotional bodies that was specific to the right hemisphere and correlated with participants’ disposition to feel personal distress. This ‘orienting’ inhibitory response to emotional bodies was also paralleled by a general drop in categorization accuracy when stimulating the right but not the left motor cortex. Conversely, at 300 ms, greater excitability for negative, positive and neutral movements was found in both hemispheres. This later motor facilitation marginally correlated with participants’ tendency to assume the psychological perspectives of others and reflected simulation of the movement implied in the neutral and emotional body expressions. These findings highlight the motor system’s involvement during perception of emotional bodies. They suggest that fast orienting reactions to emotional cues—reflecting neural processing necessary for visual perception—occur before motor features of the observed emotional expression are simulated in the motor system and that distinct empathic dispositions influence these two neural motor phenomena. Implications for theories of embodied simulation are discussed.

**Electronic supplementary material:**

The online version of this article (doi:10.1007/s00429-014-0825-6) contains supplementary material, which is available to authorized users.

## Introduction

Perceiving and reacting to the emotional states of other individuals are critical for survival. Facial and bodily expressions convey important information about another person’s feelings and intentions. Nevertheless, to date most investigations of emotion perception have focused on brain activity generated by the perception of facial expressions (see Fusar-Poli et al. 2009 and Sabatinelli et al. 2011 for meta-analyses) and neglected the body by comparison. Imaging studies have suggested that processing emotional body expressions recruits a complex neural network which includes not only visual areas, but also cortical and subcortical regions involved in emotional processing (e.g., the amygdala, anterior insula, and orbitofrontal cortex) and fronto-parietal sensorimotor regions involved in action planning and execution (de Gelder et al. 2010; Tamietto and de Gelder 2010). However, the nature of such motor activation is unclear.

According to embodied simulation theories, since covert emotional states (e.g., happiness) are often associated with overt motor behaviors (e.g., smiling, joyful body postures and gestures), observers can understand the unobservable emotional states of others by embodying their observable motor behavior through motor (or somato-motor) resonance mechanisms that tap into the motor (somato-motor) response associated with generating the perceived expression (Adolphs 2002; Gallese et al. 2004; Goldman and Sripada 2005; Keysers and Gazzola 2006, 2009; Gallese 2007; Oberman et al. 2007; Bastiaansen et al. 2009; Niedenthal et al. 2010; Gallese and Sinigaglia 2011). Most radical “motoric-centric” versions of these theories contend that motor resonance occurs prior to the activity in emotion-related regions (thus very early in time) and is necessary for assigning emotional meaning to visual signals (thus they would play a causal role in visual perception) (e.g., Carr et al. 2003; Iacoboni 2009). However, to date, these hypotheses were mainly based on the indirect imaging evidence of a co-activation of motor and emotional regions during observation of emotional expressions. Although studies suggest that portions of the motor system indeed transmit information to emotion-related regions during emotion perception (Jabbi and Keysers 2008) and that manipulation of posture and motor activity affects perception of emotions in others (Oberman et al. 2007; Niedenthal et al. 2010), whether the cortical motor system is engaged early and whether this engagement reflects resonance mechanisms necessary for visual perception remain speculative.

Support for a causal role of somatosensory (rather than motor) regions comes from neuropsychological and transcranial magnetic stimulation (TMS) studies showing that both stable lesions and transient disruption of the right somatosensory cortex impair the recognition of emotions from facial expressions (Adolphs et al. 2000; Pourtois et al. 2004; see also Banissy et al. 2011). In particular, Pitcher et al. (2008) showed this effect by administering pairs of TMS pulses early during visual perception (at 100–140 ms and 130–170 ms from stimulus onset), suggesting that the right somatosensory cortex is promptly engaged during the perception of facial expressions. However, it is unclear whether similar engagement would be critical for the recognition of body rather than facial expressions. Moreover, it is unclear whether early somatosensory (or motor) activity reflects a resonance mechanism or neural processing of another kind.

For the motor system, the picture is complicated by the fact that emotional cues may trigger fast motor reactions (Ekman and Davidson 1994; Izard 1994; Frijda 2009) rather than motor resonance. Indeed, other scholars embracing an evolutionary perspective on emotion processing have proposed that attribution of emotional value to visual stimuli occurs, at least initially, in subcortical circuits (e.g., amygdala, pulvinar, superior collicolus, etc.; Morris et al. 1999; Luo et al. 2007; Tamietto et al. 2009; de Gelder et al. 2010; LeDoux 2012). In this vein, early motor reactivity during perception of emotional bodies would reflect (non-simulative) emotionally appropriate motor reactions serving adaptive purposes (e.g., fight/flight reactions), rather than motor resonance processing necessary for visual perception (Tamietto et al. 2009; de Gelder et al. 2010; LeDoux 2012).

In the present study, we directly tested the different predictions made by simulative and non-simulative theories regarding the time course of motor system responses to emotional bodies and the potential role of such motor responses in visual perception of emotional bodies. To this aim, we used single-pulse TMS during an emotion recognition task in which participants observed and actively categorized pictures of happy, fearful and neutral body movements and static postures. TMS was administered over the right M1 (Exp1M1right) or left M1 (Exp2M1left) at two critical time points, i.e., at 150 and 300 ms from picture onset.

This paradigm allowed us to record TMS-induced motor-evoked potentials (MEPs) during perception of emotional body expressions. In this way, we non-invasively monitored changes in motor excitability that would reflect the neural responses hypothesized by embodied simulation theories (i.e., embodiment of the observed motor behavior, that is, motor resonance) or other types of responses subserving perception (e.g., orienting responses) or body survival (e.g., freezing or fight/flight reactions). Indeed, measurement of MEPs is a well-established approach to exploring motor resonance in humans (Fadiga et al. 1995, 2005; Avenanti et al. 2007, 2013b; Aglioti et al. 2008; Urgesi et al. 2010; Candidi et al. 2010; Catmur et al. 2011) and a number of studies have also shown that perceptually salient and emotional stimuli affect motor excitability (Farina et al. 2001; Oliveri et al. 2003; Makin et al. 2009; Serino et al. 2009). TMS seems, therefore, to be a valuable tool for assessing the interplay between action and emotion processing within the motor system.

Importantly, since TMS pulses disrupt neural activity in the targeted regions, we also tested whether stimulation of M1 at 150 or 300 ms after stimulus onset affected visual perception of body expressions. This allowed us to combine correlational and causal approaches to test the role of the motor system in the perception of body expressions.

The early time point (150 ms) was chosen to explore possible short-latency motor responses to emotional bodies and was based on the idea that complex visual scenes including facial and contextual emotional cues modulate visual event-related potentials (ERPs) in the 100–200 ms range (Vuilleumier and Pourtois 2007; Olofsson et al. 2008) and motor excitability at 150 ms (Borgomaneri et al. 2013). Moreover, this timing fits with the time window tested by Pitcher et al. (2008). If emotional body perception is associated with fast motor reactions to emotional body cues, we might expect differential excitability for emotional and non-emotional movements at this latency and stronger reactivity in the right hemisphere, which may be dominant for emotional processing (Borod 2000). The later time point (300 ms) was chosen based on action observation studies showing that neural activity reflecting motor resonance is typically detected at about 250–350 ms after stimulus onset in the motor cortices (Nishitani et al. 2004; Catmur et al. 2011; Barchiesi and Cattaneo 2013) and on the finding that observation of emotional and non-emotional movements induces motor resonance in the observer’s left M1 at 300 ms after stimulus onset (Borgomaneri et al. 2012). Thus, at this time point we expected neural activity reflecting the encoding of the motor features of observed actions, independent of their emotional meaning (as found in Borgomaneri et al. 2012 for the left M1).

Since studies suggest that participants with a greater tendency to take the psychological perspective of another may show stronger resonant activations (Gazzola et al. 2006; Cheng et al. 2008; Avenanti et al. 2009a; Minio-Paluello et al. 2009; Martínez-Jauand et al. 2012; Schaefer et al. 2012) and different empathy traits may modulate neural activity during social perception (Singer et al. 2004; Lamm et al. 2007, 2010; Melloni et al. 2013; Borgomaneri et al. 2013; Bufalari and Ionta, 2013), we explored the relation between changes in motor excitability and individual scores of dispositional empathy using the Interpersonal Reactivity Index (IRI) (Davis 1996).

If early M1 reactivity reflects pure motor reactions to emotional cues that are epiphenomenal for visual recognition (as suggested by non-simulative theories), whereas later motor resonance plays an active role in perception (as suggested by embodied simulation theories), we might expect that M1 stimulation at 300 ms but not at 150 ms from stimulus onset would disrupt participants’ performance in the emotion recognition task. Conversely, if early motor activity reflects neural processing necessary for perceiving body expressions, whereas motor resonance at 300 ms reflects an embodiment of the observed expression occurring after its visual recognition, we might expect that M1 stimulation at 150 ms but not at 300 ms would impair task performance.

## Materials and methods

### Participants

Fifty-six healthy subjects took part in the study. Twenty participants (10 men, mean age ± SD: 23.7 years ± 2.4) were randomly assigned to Experiment 1 in which the right M1 was stimulated (Exp1M1right) and other 20 (9 men, 23.7 years ± 1.6) to Experiment 2 in which the left M1 was stimulated (Exp2M1left). A further group of 16 participants (7 men, 25.5 years ± 3.1) took part in a third control experiment in which sham stimulation was performed (Exp3Sham). The experiments were carried out at the Centro studi e ricerche in Neuroscienze Cognitive, Department of Psychology, University of Bologna. All participants were right-handed according to a standard handedness inventory (Oldfield 1971) and free from any contraindication to TMS (Rossi et al. 2009). They gave their written informed consent to take part in the study, which was approved by the local ethics committee and carried out according to the Declaration of Helsinki. No discomfort or adverse effects during TMS were reported or noticed.

### Visual stimuli

**Fig. 1:**
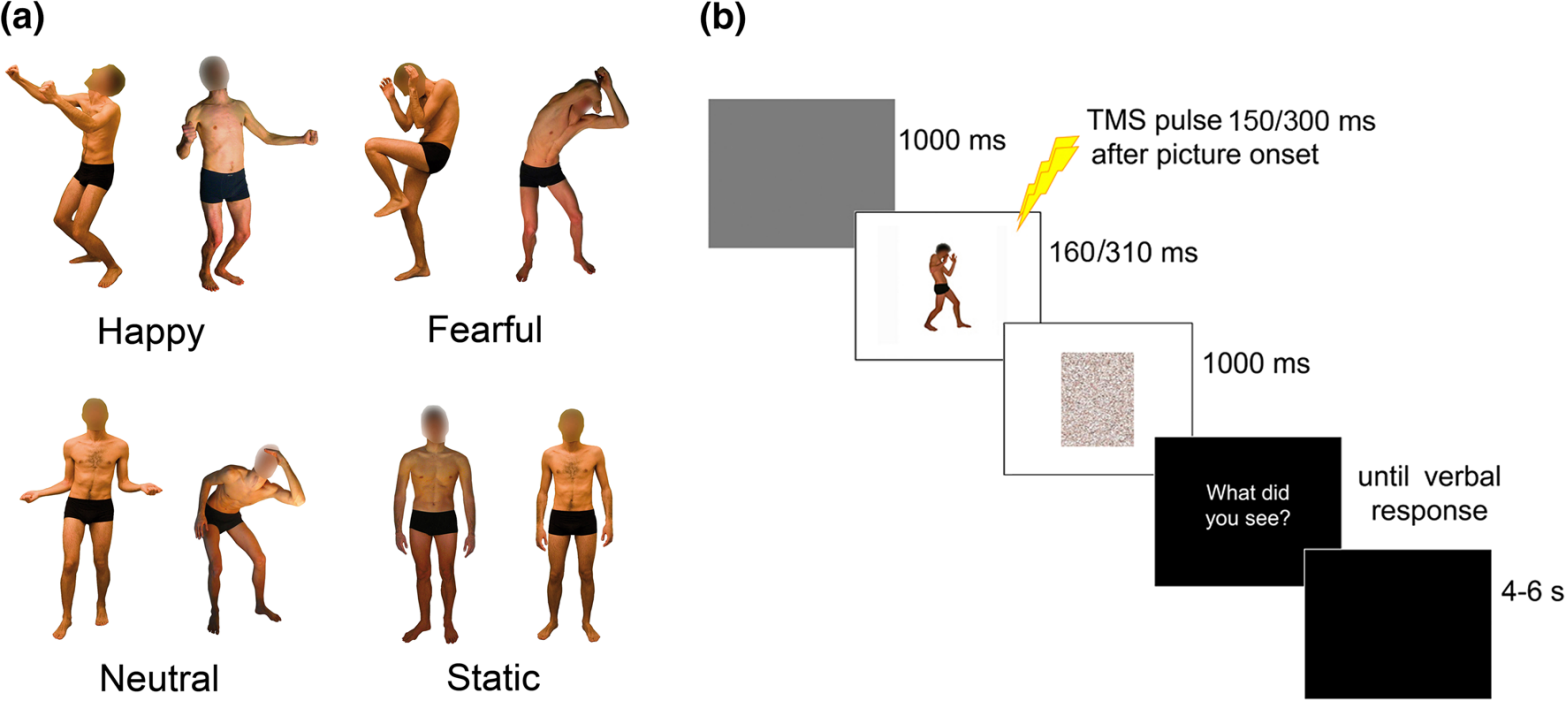
**a** Examples of visual body stimuli. **b** Trial sequence

In all the experiments, different types of pictures were presented on a 19-inch screen located 80 cm away from the participants. Sixty pictures were selected from a validated database (Borgomaneri et al. 2012). Pictures depicted four different actors in emotional and neutral postures (Fig. 1a). To focus specifically on body-related information, the face was blanked out in all the pictures. Stimuli included pictures of emotionally positive (happy) and negative (fearful) movements, neutral movements (i.e., actions with implied movement comparable to emotional body expressions but with no emotional meaning) and static neutral postures (baseline).

During the recording of neutral movements, instructions to the actors specified the action to be performed (e.g., jump rope). For emotional expressions, instructions specified a familiar scenario (e.g., you have just won the lottery) or involved a potential threat (e.g., a tennis ball was thrown at the actor). Stimuli were selected from an initial sample of about 1,000 images based on two pilot studies in which emotional ratings and emotion recognition data were collected, resulting in a final selection of 15 fearful body expressions, 15 happy body expressions, 15 neutral movements and 15 static postures that were well recognized as prototypical representations of the different expressions (see Borgomaneri et al. 2012 for details). All the emotional and neutral movement stimuli represented a whole-body movement with a clear involvement of upper limbs (implied motion stimuli). In none of the stimuli did the model interact with objects or other individuals. To rule out that any differential modulatory effect in the left and right M1 was due to a different amount of implied motion of the models’ left or right hands, mirror-reflected copies of the selected stimuli were also created. In each experiment, half the participants were tested with the original version of the stimuli, and the remaining half were tested with mirror-reflected copies.

### Transcranial magnetic stimulation and electromyography recording

Both Exp1M1right and Exp2M1left started with the electrode montage setup, detection of optimal scalp position and measurement of resting motor threshold. To explore motor excitability, MEPs induced by TMS were recorded from the first dorsal interosseus (FDI) muscles with a Biopac MP-35 (Biopac, USA) electromyograph. In Exp1M1right and Exp2M1left, MEPs were recorded from the left and the right FDI, respectively (contralateral to the stimulated hemisphere). To check muscle relaxation during MEP recording, EMG activity was also recorded from the FDI muscle ipsilateral to the stimulated hemisphere. EMG signals were band-pass filtered (30–500 Hz), sampled at 5 kHz, digitized and stored on a computer for off-line analysis. Pairs of silver-chloride surface electrodes were placed in a belly-tendon montage with ground electrodes on the wrist. A figure-of-eight coil connected to a Magstim Rapid2 stimulator (Magstim, Whitland, Dyfed, UK) was placed over M1. The intersection of the coil was placed tangentially to the scalp with the handle pointing backward and laterally at a 45° angle from the midline. With this antero-medial coil orientation, biphasic TMS pulses elicited eddy currents in the brain flowing in a posterior–anterior/anterior–posterior direction approximately perpendicular to the line of the central sulcus. The chosen coil orientation may require slightly greater (biphasic) pulse intensity to elicit MEPs relative to the opposite postero-lateral coil orientation (Kammer et al. 2001). However, the antero-medial orientation is best suited for recording MEPs during visual tasks because it does not require the experimenter holding the coil to stand in front of the participant during TMS.

Detection of optimal scalp position and resting motor threshold was performed as follows. Using a slightly suprathreshold stimulus intensity, the coil was moved over the target hemisphere to determine the optimal position from which maximal amplitude MEPs were elicited in the contralateral FDI muscle. The optimal position of the coil was then marked on the scalp with a pen to ensure correct coil placement throughout the experiment. In Exp1M1right and Exp2M1left, the intensity of magnetic pulses was set at 125 % of the resting motor threshold (rMT), defined as the minimal intensity of stimulator output that produces MEPs with an amplitude of at least 50 μV from the muscle with 50 % probability (using about 20 pulses). Mean stimulation intensity (mean % of maximal stimulator output ± SD) was statistically comparable in Exp1M1right (72.0 ± 10.2 %) and Exp2M1left (67.7 ± 5.2 %; *t*(38) = 1.70, *p* = 0.1). The absence of any voluntary contractions was visually verified continuously throughout the experiments. When muscle tension was detected the experiment was briefly interrupted and the subject was invited to relax.

In Exp3Sham, no electrophysiological preparation was necessary since no EMG signal was recorded. Sham stimulation was performed by placing the coil tilted at 90° over the vertex. In all subjects, stimulation intensity was set at 70 % of the maximal stimulator output, corresponding to the mean intensity used in Exp1M1right and Exp2M1left. Although no current was induced in the brain, sham TMS produced some scalp sensations and auditory clicks comparable to active stimulation.

### Procedure and experimental design

The experiments were programmed using Matlab software to control picture presentation and to trigger TMS pulses. In Exp1M1right and Exp2M1left, MEPs were collected in two separate blocks of 60 trials. In each block, subjects performed an emotion recognition task: they were presented with a picture and were asked to categorize it as either a happy, fearful, neutral dynamic or static body posture. Trial sequence was as follows: a gray screen (1 s duration) indicated the beginning of the trial, and it was followed by the test picture projected at the center of the screen (Fig. 1b). In half the trials, stimuli were presented for 160 ms and TMS was delivered at 150 ms from stimulus onset. In the remaining trials, stimuli were presented for 310 ms and TMS was delivered at 300 ms from stimulus onset. Stimulus duration was randomly distributed in the two blocks. The picture was followed by a random-dot mask (obtained by scrambling the corresponding sample stimulus by means of custom-made image segmentation software) lasting 1 s. Then the question “What did you see?” appeared on the screen, and the subject provided a verbal response (forced choice). Possible choices were: happy, fearful, neutral, static. An experimenter collected the answer by pressing a computer key. To avoid changes in excitability due to verbal response (Tokimura et al. 1996), participants were invited to answer only during the question screen, a few seconds after the TMS pulse (Tidoni et al. 2013). After the response, the screen appeared black for 4–6 s, ensuring an inter-pulse interval greater than 10 s and thereby avoiding changes in motor excitability due to TMS per se (Chen et al. 1997). To reduce the initial transient-state increase in motor excitability, before each block two magnetic pulses were delivered over the targeted M1 (inter-pulse interval >10 s). Each block lasted about 10 min.

To provide control conditions for comparing behavioral performance in Exp1M1right and Exp2M1left, we carried out Exp3Sham in which sham TMS was performed instead of active M1 stimulation. In this third control experiment, the stimuli, the task and the trial structure were the same as in Exp1M1right and Exp2M1left, but no MEPs were recorded. Only behavioral performance on the emotion recognition task was measured.

### Subjective measures

After TMS, only subjects in Exp1M1right and Exp2M1left were presented with all the stimuli (shown in a randomized order) and asked to judge arousal, valence and perceived movement using a 10 cm electronic visual analogue scale (VAS). To avoid building up artificial correlations between the different judgments, each rating was collected separately during successive presentations of the whole set of stimuli (Avenanti et al. 2009a). Finally, subjects completed the IRI questionnaire (Davis 1996), a 28-item self-report survey that consists of four subscales, namely Perspective Taking (PT, which assesses the tendency to spontaneously imagine and assume the cognitive perspective of another person), Fantasy Scale (FS, which assesses the tendency to imaginatively transpose oneself into fictional situations), Empathic Concern (EC, which assesses the tendency to feel sympathy and compassion for others in need) and Personal Distress (PD, which assesses the extent to which an individual feels distress in emotional interpersonal contexts). PT and FS assess cognitive components of empathy, while EC and PD correspond to other-oriented empathy reactions and self-oriented emotional distress, respectively (Davis 1996).

### Data analysis

Neurophysiological and behavioral data were processed off-line. Mean MEP amplitudes in each condition were measured peak-to-peak (in mV). MEPs associated with incorrect answers were discarded from the analysis (<6 %). Since background EMG is known to affect motor excitability (Devanne et al. 1997), MEPs with preceding background EMG deviating from the mean by more than 2 SD were removed from further analysis (<6 %). To compare motor excitability in Exp1M1right and Exp2M1left we computed MEP contrast indices by subtracting the mean MEP amplitudes recorded in the static body posture condition from the MEP amplitudes recorded in the three dynamic conditions (happy, fearful, neutral movements). MEP contrasts (dynamic-static) were first analyzed by means of a three-way mixed model ANOVA with Area (2 levels: Exp1M1right and Exp2M1left) as a between-subjects factor, and Time (2 levels: 150 and 300 ms) and Movement type (3 levels: happy, fearful and neutral) as within-subjects factors.

To test whether the TMS pulse had interfered with visual recognition of body expressions, we compared behavioral performance in the emotion recognition task across the three experiments. Accuracy (i.e., % correct responses) was analyzed by means of a mixed model two-way ANOVA with Area (3 levels: Exp1M1right, Exp2M1left and Exp3Sham) as a between-subjects factor, and Time (2 levels: 150 and 300 ms) as a within-subjects factor. A preliminary ANOVA that also included the factor Movement type (see Supplementary Table 1) did not reveal any interaction between Movement type and Area (*p* > 0.27), so data were collapsed across the Movement type factor. Mean VAS ratings for arousal, valence and implied movement were analyzed by means of mixed model two-way ANOVAs with the factors Area (2 levels: Exp1M1right and Exp2M1left) and Movement type (4 levels: happy, fearful, neutral and static). Because subjective ratings in the various experimental conditions were slightly correlated (−0.16 < *r* < 0.50, with Pearson coefficients computed across the experiments), and therefore not independent we then corrected the *p*-level for the number of ANOVAs. In all the ANOVAs, post-hoc comparisons were carried out by means of the Newman–Keuls test. Finally, to test the relation between behavioral performance, dispositional empathy and motor excitability, standard regression and correlational analyses were performed. In these analyses, MEP contrasts were entered as dependent variables, whereas indices of performance accuracy in the emotion recognition task (accuracy drop contrast: mean % accuracy at 150 ms−mean % accuracy at 300 ms) and the four subscales of the IRI questionnaire were entered as predictors.

## Results

### Subjective measures

The Area × Movement type ANOVAs carried out on valence, arousal and implied motion scores showed only a main effect of Movement type (all *F* > 123.43, *p* < 0.0001). No other main effects or interactions were significant in the ANOVAs (all *p* > 0.43; see Table 1).

**Table 1:** Mean ± standard deviation subjective evaluations (arousal, valence and perceived implied motion) of stimuli used in the first (Exp1M1right) and the second experiment (Exp2M1left)

	Static	Happy	Neutral	Fearful
Exp1M1right				
Arousal	1.50 ± 1.32	5.84 ± 1.49	3.91 ± 1.86	6.13 ± 1.25
Valence	4.71 ± 0.22	8.04 ± 0.83	5.16 ± 0.55	1.52 ± 0.71
Perceived motion	0.46 ± 0.44	6.03 ± 1.61	5.96 ± 1.18	5.10 ± 1.51
Exp2M1left				
Arousal	1.04 ± 1.17	5.56 ± 1.70	3.46 ± 1.79	6.32 ± 1.15
Valence	4.82 ± 0.16	7.87 ± 0.93	5.27 ± 0.53	1.42 ± 0.75
Perceived motion	0.42 ± 0.44	6.09 ± 1.57	5.98 ± 1.45	5.18 ± 1.93

Post-hoc analyses showed that valence ratings were lower for fearful movements relative to happy and neutral movements and static body postures (all *p* < 0.001); moreover, valence ratings were higher for happy relative to neutral movements and static postures (all *p* < 0.001); neutral movements were considered more positive than static postures (*p* = 0.004). Arousal scores were greater for happy and fearful movements relative to neutral movements and static postures (all *p* < 0.001). Moreover, arousal ratings were not significantly different between fearful and happy movements (*p* = 0.07) whereas neutral movements were considered more arousing than static postures (*p* < 0.001). Implied motion scores were greater for happy, neutral and fearful movements relative to static postures (all *p* < 0.001); moreover, scores were higher for happy and neutral movements relative to fearful movements (all *p* < 0.002). Happy and neutral movements contained the same amount of implied motion (*p* = 0.69).

### Behavioral performance in the emotion recognition task

**Fig. 2:**
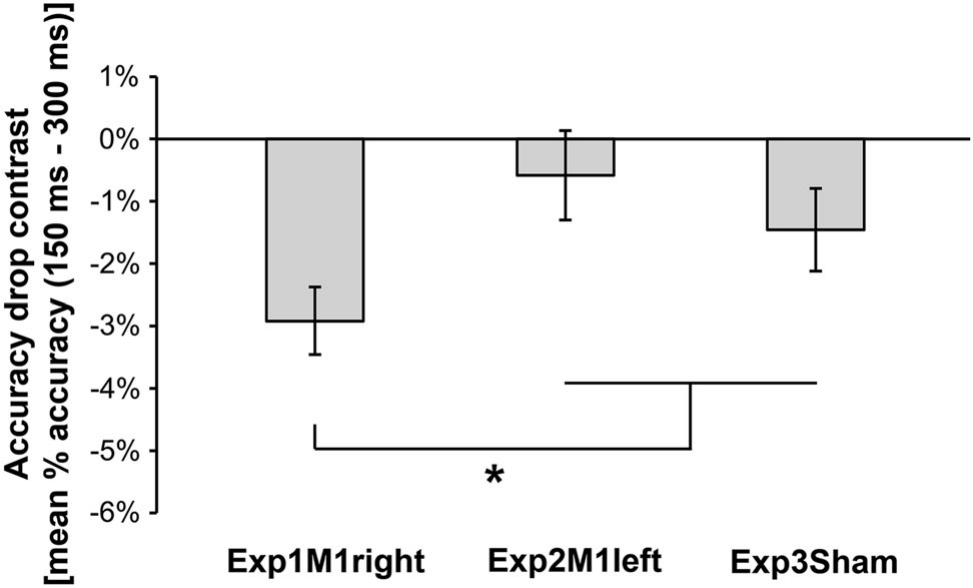
Effect of TMS on recognition accuracy. Accuracy drop contrast (mean drop in % accuracy found in the early relative to the late temporal condition) computed in the three experiments. Only in Exp1M1right there was a significant drop in accuracy in the early temporal condition (see main text). The drop in accuracy detected in Exp1M1right was greater than that found in the other two experiments. *Error bars* indicate s.e.m. *Asterisks* denote significant comparisons (*p* < 0.05)

The ANOVA on accuracy data showed a main effect of Time (*F*(1,53) = 19.50, *p* < 0.0001) and, importantly, a significant Time × Area interaction (*F*(2,53) = 3.57, *p* = 0.035). This was accounted for by lower accuracy in the early (150 ms) relative to the late (300 ms) temporal condition (mean % of correct responses ±SD: 92.8 % ± 4.0 vs 95.8 % ± 2.9, *p* = 0.0006) found in Exp1M1right only. Indeed, the same comparison between temporal conditions was not significant in Exp2M1left (94.1 % ± 4.7 vs 94.7 % ± 3.8; *p* = 0.37) or Exp3Sham (94.1 % ± 2.3 vs 95.5 % ± 2.8; *p* = 0.12). These data indicate that in Exp1M1right there was a small but significant drop in accuracy in the 150 ms relative to the 300 ms condition (−2.9 % ± 2.4; Fig. 2), whereas the drop was not significant in Exp2M1left (−0.6 % ± 3.2) or Exp3Sham (−1.5 % ± 2.7). Planned comparisons also showed that the accuracy drop was greater in Exp1M1right than in the other two experiments (*p* = 0.018; Fig. 2) which in turn did not differ from one another (*p* = 0.39). These findings suggest that TMS administered over right M1 at 150 ms from stimulus onset selectively interfered with visual recognition of body expressions. This interference was similar across body expressions (see Supplementary Table 1).

### Neurophysiological data

**Fig. 3:**
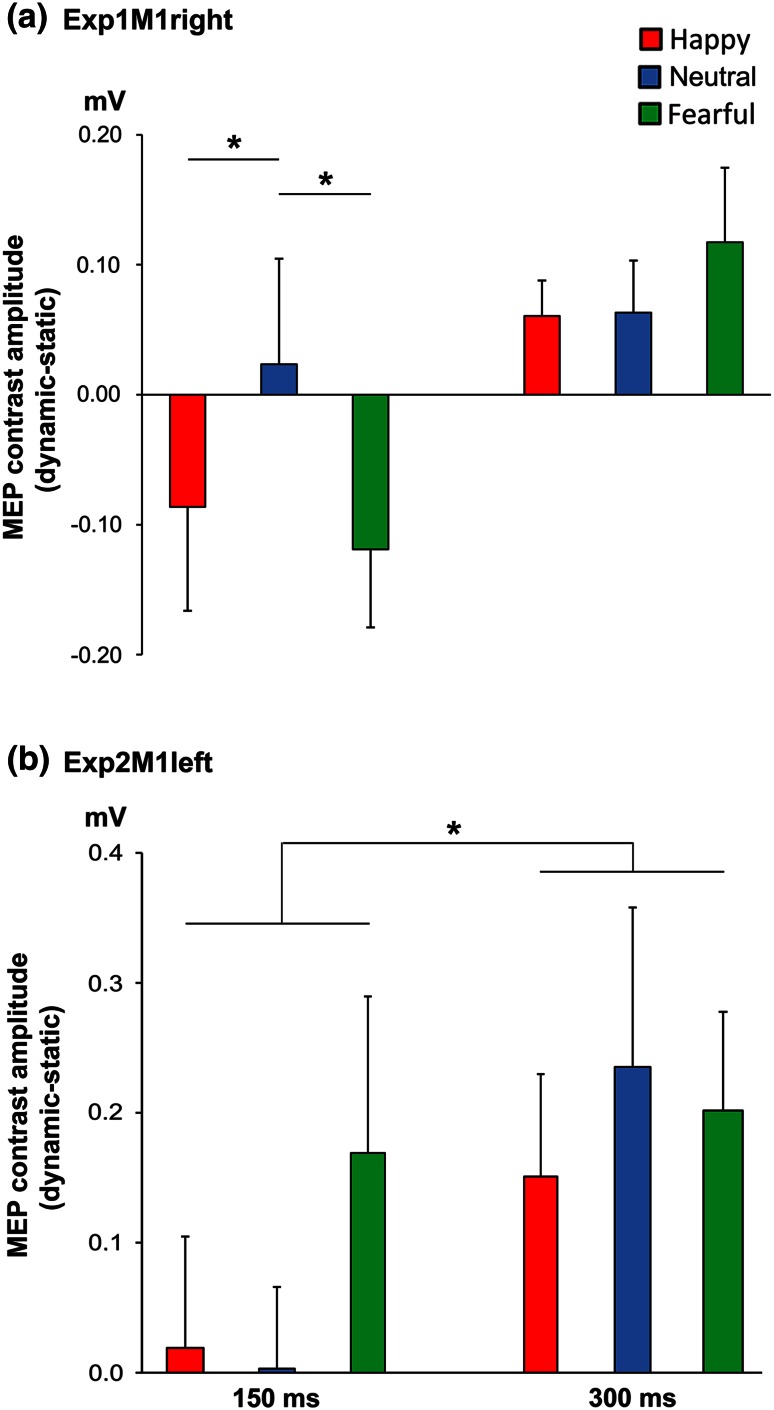
Neurophysiological modulations during the emotion recognition task. MEP amplitude contrasts (dynamic–static) during perception of happy, neutral and fearful body postures at 150 and 300 ms from the stimulus onset. **a** Data from the first (Exp1M1right) experiment showing an early suppression of MEPs for emotional bodies and a later increase of MEPs for the three dynamic expressions. **b** Data from the second experiment (Exp2M1left), showing greater MEPs for the three dynamic expressions in the late relative to the early temporal condition. See main text for further statistical results. *Error bars* indicate s.e.m. *Asterisks* denote significant comparisons (*p* < 0.05)

The Area × Time × Movement type ANOVA on MEP contrasts (happy-static, fearful-static and neutral-static) showed a significant triple interaction (*F*(2,76) = 3.67, *p* = 0.03). This interaction seems to be driven by the fact that the MEP suppression obtained when viewing emotional (happy and fearful) compared to neutral bodies, which is only significant at 150 ms in the right hemisphere (Fig. 3a), decreases from 150 ms to 300 ms in the right hemisphere, while the opposite trend occurs in the left hemisphere {[mean (happy and fearful) − neutral]_150M1right _− [mean (happy and fearful) − neutral]_300M1right_ > [mean (happy and fearful) − neutral]_150M1left _− [mean (happy and fearful) − neutral]_300M1left_; two sample *t* test, *p* = 0.03)}.

To further explore the triple interaction we carried out two separate ANOVAs, one for each Area. The Time × Movement type ANOVA on MEP contrasts from Exp1M1right showed a Time × Movement type interaction (*F*(2,38) = 3.35, *p* = 0.046). The post-hoc analysis showed that when TMS was administered at 150 ms after stimulus onset, observation of happy and fearful expressions brought about lower MEP values relative to observation of neutral movements (*p* = 0.049 and *p* = 0.03, respectively), indicating a reduction of motor excitability for emotional body stimuli. This inhibitory response was comparable for emotionally positive and negative body expressions (*p* = 0.55).

In contrast, when TMS was administered at 300 ms after stimulus onset, MEPs were facilitated in a similar way during observation of emotional and neutral movements (all comparisons *p* > 0.32). No other effects were significant in the ANOVA (all *F* < 2.33, *p* > 0.14; Fig. 3a).

The Time × Movement type ANOVA on MEPs recorded in Exp2M1left showed a main effect of Time (*F*(1,19) = 4.65, *p* = 0.044) but no main effect of Movement type or Time × Movement type interaction (all *F* < 1.46, *p* > 0.24). MEPs were larger at 300 ms relative to those recorded at 150 ms from stimulus onset (Fig. 3b).

To specifically test whether observation of emotional and non-emotional movements induced motor resonance, a series of planned comparisons were performed. These showed that, collapsing across hemispheres, MEPs recorded at 300 ms during observation of happy (mean amplitude ± SD: 1.52 mV ± 0.92), fearful (1.58 mV ± 0.96) and neutral movements (1.57 mV ± 1.00) were larger than those recorded when seeing static body postures (1.42 mV ± 0.75, all comparisons *p* < 0.03), indicating that seeing emotional and neutral implied motion stimuli brought about an increase in motor excitability relative to static controls. These motor facilitations for emotional and neutral movements were comparable in the two hemispheres (all *p* > 0.19).

To further test motor excitability in the early time window an additional analysis was performed. A previous study showed that seeing emotionally negative scenes increases the excitability of the left M1 at 150 ms after stimulus onset (Borgomaneri et al. 2013). Although the Time × Movement type interaction was not significant in Exp2M1left, visual inspection of the MEPs in Fig. 2b suggests a possible increase in M1 activity for fearful expressions in the 150 ms condition. However, a planned *t*-test comparing fearful with neutral movements at 150 ms revealed only a non-significant trend (*p* = 0.067).

### Relation between changes in motor excitability and behavioral performance

**Fig. 4:**
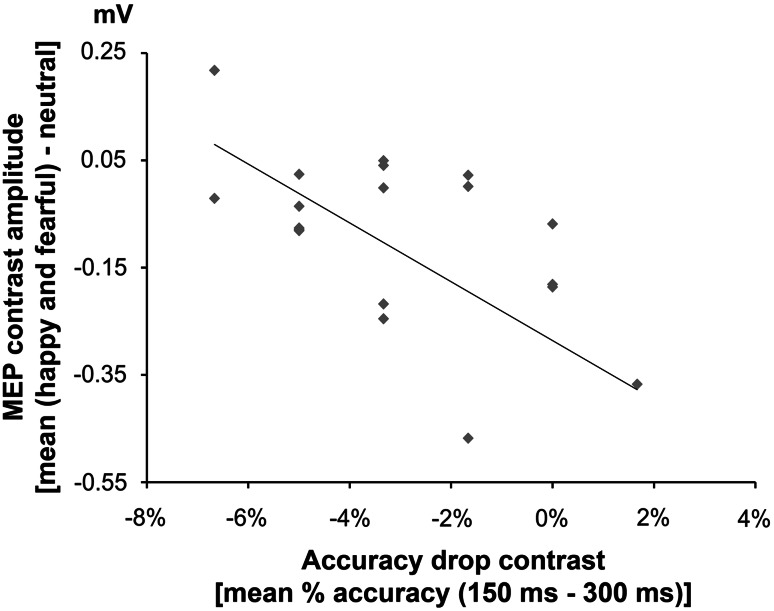
Simple correlation between neurophysiological and behavioral changes in Exp1M1right. MEP contrast index, representing the early changes in motor excitability (mean amplitude during happy and fearful body postures minus mean amplitude during neutral body postures) significantly correlated with the index representing the early interferential effect of right M1 stimulation on visual recognition of body expressions, i.e., the accuracy drop contrast (mean drop in % accuracy found in the early relative to the late temporal condition of Exp1M1right)

In sum, we found that seeing both emotionally positive and negative movements reduced motor excitability at 150 ms relative to neutral movements in Exp1M1right. No similar modulation of motor excitability was found in Exp2M1left at the same temporal delay. In addition, behavioral performance suggests an accuracy drop for the early temporal condition only in Exp1M1right. To test for a relation between the neurophysiological and behavioral data, we computed a simple correlation between the MEP contrast at 150 ms [mean (happy and fearful) − neutral movement] and an index expressing the drop in accuracy in the early timing [accuracy drop contrast: (average % accuracy at 150 ms) − (average % accuracy at 300 ms)]. We found that the MEP contrast was strongly and negatively correlated with the accuracy drop contrast found in Exp1M1right (*r* = −0.57, *p* = 0.008; Fig. 4), with a stronger inhibitory response associated with a smaller drop in accuracy and less inhibition with a greater accuracy drop. The same analysis conducted on Exp2M1left was not significant (*r* = 0.12, *p* = 0.61). These findings suggest a close link between visual recognition of body expressions and early changes in the excitability of the right M1.

### Relation between changes in motor excitability and dispositional empathy

While early motor reactivity in the right hemisphere consisted of a reduction in excitability for emotional bodies, we found a motor facilitation for all dynamic bodies at 300 ms. This motor facilitation was comparable for emotional and neutral movements and was similar in the two hemispheres.

To test whether these two neurophysiological effects were related to individual differences in dispositional empathy, two multiple regression analyses were carried out. MEP contrasts, computed based on the results of the ANOVAs, were entered as dependent variables in the regression models, and individual scores from the IRI subscales (Fantasy, Perspective Taking, Empathic Concern and Personal Distress) were entered as predictors.

**Fig. 5:**
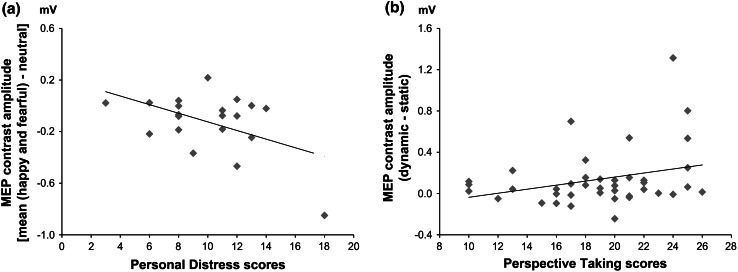
Simple correlations between early and late neurophysiological effects and personality dispositions. **a** Simple correlation between early MEP contrasts in Exp1M1right (mean amplitude during happy and fearful body postures minus mean amplitude during neutral body postures) and the Personal Distress subscale of the Interpersonal Reactivity Index. **b** Simple correlation between late MEP contrasts in Exp1M1right and Exp2M1left (mean amplitude during dynamic body postures minus mean amplitude during static body postures) and the Perspective Taking subscale of the Interpersonal Reactivity Index

In the first analysis we considered the contrast representing the early inhibition found in Exp1M1right [mean (happy and fearful) − neutral movement]. The regression model was non-significant (*R* ^2^ = 0.27, *F*(4,15) = 1.41, *p* = 0.28; no statistical outliers with residual >2 sigma were present in the data set). However, personal distress (PD) was a significant negative predictor of the emotion-related MEP change (*β* = −0.52, *t*(15) = −2.33, *p* = 0.03), showing greater MEP reduction in participants with higher PD scores. No other predictors were significantly related to the neurophysiological index. Simple correlations confirmed that emotion-related MEP reduction correlated with PD (*r* = −0.49, *p* = 0.029; Fig. 5a) but not with other IRI subscales (all *p* > 0.77). These findings suggest that subjects who scored high in PD showed greater early motor inhibition in the right hemisphere when seeing emotional bodies.

Since early motor reactivity in Exp1M1right correlated with both PD scores and behavioral performance (see previous paragraph), we also explored the relation between these two variables as a control analysis. The simple correlation was not significant in this case (*r* = −0.01, *p* = 0.96), suggesting that inter-individual differences in PD scores were not associated with the magnitude of the interferential effect of right M1 stimulation. Additionally, we computed a regression model in which PD scores and the accuracy drop index were entered as predictors of early motor reactivity. The regression was significant (*R* ^2^ = 0.57, *F*(2,17) = 11.49, *p* = 0.0007; no statistical outliers with residual >2 sigma were present in the data set) and both PD scores (*β* = −0.50, *t*(17) = −3.14, *p* = 0.006) and the drop in accuracy (*β* = −0.58, *t*(17) = −3.67, *p* = 0.002) were significant independent predictors of early motor reactivity.

Finally, we tested whether the bilateral motor facilitation we found at 300 ms for emotional and neutral movements was related to dispositional empathy. Since participants in Exp1M1right and Exp2M1left showed very similar motor responses to dynamic stimuli and scored similarly on all the IRI subscales (all *p* > 0.49), we pooled the two groups together to increase statistical power. A MEP modulation index reflecting the late motor facilitation for dynamic bodies was computed by averaging the MEP contrasts computed for happy, fearful and neutral movements at 300 ms [mean (happy, neutral and fearful) − static]. This index was entered as a dependent variable in a standard regression model and the IRI subscales were entered as predictors. The regression model was non-significant [*R* ^2^ = 0.11, *F*(4,35) = 1.05, *p* = 0.39; no statistical outliers with residual >2 sigma were present in the data set], and no predictors were found to be significant (all *p* > 0.16). Based on previous studies showing a relation between cognitive empathy and imitative behavior (Chartrand and Bargh 1999) and motor resonance (Gazzola et al. 2006; Keysers and Gazzola 2006; Cheng et al. 2008; Avenanti et al. 2009a) we specifically tested the bivariate relation between late motor facilitation and scores on the IRI Perspective Taking (PT) subscale. The Pearson coefficient showed a marginally significant positive correlation (*r* = 0.30, *p* = 0.06; Fig. 5b). This suggests that individuals who reported higher levels of PT tended to show stronger motor resonance when seeing emotional and neutral movements.

## Discussion

It is well established that the motor system is recruited during emotion processing (Lang 1993; Ekman and Davidson 1994; Izard 1994; Frijda 2009). However, the nature of motor cortex activations in the perception of emotional body language is a matter of debate. According to embodied simulation accounts, neural activity in the observer’s motor system reflects motor resonance, i.e., simulation of the motor features of the observed emotional expression (Carr et al. 2003; Leslie et al. 2004; Oberman et al. 2007; Bastiaansen et al. 2009; Niedenthal et al. 2010; Gallese and Sinigaglia 2011). On the other hand, early motor reactivity may reflect different non-simulative processing, including fast motor reactions to emotional cues (i.e., fight/flight reactions) or neural processing that facilitates visual perception (e.g., orienting responses) (Tamietto et al. 2009; de Gelder et al. 2010; LeDoux 2012). Here we tested the hypothesis that motor responses to emotional cues and motor resonance are both implemented in the motor system but at different times. We found that seeing emotional body movements reduced MEP amplitude at 150 ms, only after stimulation of the right M1. This early inhibition of motor excitability, which may reflect an orienting response toward emotional cues, was comparable for fearful and happy expressions and larger than for neutral movements. Moreover, at 150 ms, TMS over right M1 interfered with accuracy in the emotion recognition task. No similar effects were found with sham or left M1 stimulation. Greater TMS interference on task accuracy correlated with reduced changes in motor excitability, suggesting a link between neural activity reflecting early orienting and visual recognition of body expressions. In addition, orienting responses correlated with the participants’ scores on the Personal Distress scale of the IRI.

At 300 ms, greater MEP amplitudes were measured for negative, positive and emotionally neutral movements relative to static body postures in both hemispheres. This later increase in motor excitability indexed the presence of body motion in the stimulus rather than its emotional content. Indeed, MEP facilitation was comparable for the three dynamic conditions and possibly reflected motor simulation of the body movements implied in the pictures. The magnitude of this putative simulative response marginally correlated with the IRI Perspective-Taking subscale.

Our findings reveal two possibly distinct functional stages of motor cortex involvement during perception of emotional body language: an initial stage (~150 ms) reflecting early orienting responses that would actively support visual recognition of body expressions; and a later stage (~300 ms) in which the motor cortex implements resonance to any observed movements independent of their emotional content. Moreover, our study shows that distinct personality traits influence these two neural phenomena. These results shed new light on the temporal relation between the motor processes hypothesized by simulative and non-simulative theories of emotion processing and their causal role in perception. In particular, our study demonstrates that early motor activity is critical for visual perception of body expressions but this motor activity appears to reflect an orienting response rather than motor resonance.

These findings may provide some support to general proposals of embodied simulation suggesting that motor (and somato-motor) activity facilitates social and emotion perception. However, they speak against theoretical accounts that have maintained that motor resonance is an early and necessary step for the attribution of emotional meaning to visual signals (Carr et al. 2003; Iacoboni 2009). Indeed, we provide evidence that motor resonance occurs in M1 after the signals discriminating between emotional and non-emotional bodies (i.e., reflecting the orienting response) have already been processed, suggesting that at this level motor resonance may not be a prerequisite for processing the emotional features of body expressions. These findings have implications for constraining embodied simulation theories of emotion perception.

### Early orienting supporting visual perception in the right motor cortex

The major point of novelty in our study is the demonstration that the motor system is transiently modulated during perception of emotional body language, with an early and transient suppression of motor excitability in the right M1. This motor modulation reflects neural signals discriminating between emotional and non-emotional bodies and, remarkably, these signals appear critical for visual perception of body expressions.

The early right M1 modulation occurred at 150 ms, thus not only before the occurrence of the neural signature of motor resonance (i.e., the increase in motor excitability for dynamic bodies that we detected at 300 ms after stimulus onset), but even before the typical latency of occipito-temporal components of ERPs, which are supposed to reflect the visual processing underlying the structural encoding of bodies (i.e., the N170/N190 component; Gliga and Dehaene-Lambertz 2005; Thierry et al. 2006; Righart and de Gelder 2007). Previous studies have shown that, relative to neutral actions, fearful body expressions affected the ERP response in the earliest stage of visual perception, i.e., the P1 component (van Heijnsbergen et al. 2007). This occipital component, which typically occurs in the 100–150 ms window, also showed sensitivity to emotional congruence of the body and the face (Meeren et al. 2005). Our study significantly expands these previous ERP findings on several fronts. First, it shows that, in approximately the same temporal window, the brain response to emotional bodies involves not only the visual cortex but also motor structures. Second, it characterizes one of the components of such brain responses as an inhibitory modulation of the right M1. Third, it shows that such modulation is detected not only for emotionally negative but also for positive body expressions. Fourth, it shows that the magnitude of the inhibitory response correlates with the disposition to feel personal distress. Lastly, this response appears to reflect neural processing causally involved in the visual perception of body expressions.

We suggest that this early inhibitory modulation reflects the motor counterpart of an orienting response toward a salient stimulus, like an emotional body, that would manifest as a fast inhibition of the motor response. In support of this interpretation are TMS studies showing that freezing-like inhibitory modulations of M1 are detected when processing salient stimuli in different modalities, including noxious stimuli (Tamburin et al. 2001; Farina et al. 2001; Urban et al. 2004), loud acoustic stimuli (Furubayashi et al. 2000), salient auditory stimuli presented close to the body (Serino et al. 2009; Avenanti et al. 2012a), unexpected visual flashes (Cantello et al. 2000), approaching visual stimuli (Makin et al. 2009) and visual stimuli depicting pain in others (Minio-Paluello et al. 2006; Fecteau et al. 2008; Avenanti et al. 2009b). Our study adds to these previous findings by showing that during observation of emotional body language, early modulations in the right M1 are not an epiphenomenon of perception. Rather, they appear to play an active and causal role in the visual recognition of body expressions, as evidenced by the small but significant drop in task accuracy found in Exp1M1right (but not in Exp2M1left or Exp3Sham) and the close relation between the magnitude of TMS interference and orienting responses.

It should be noted that our paradigm was optimized for assessing motor excitability during accurate perception of emotional body language. For this reason, on each trial we delivered TMS at the end of picture presentation, collected expression recognition data, and considered only MEPs associated with correct recognition (Borgomaneri et al. 2012, 2013). This means, however, that the two temporal conditions (150 and 300 ms) differed not only in the latency of the TMS pulse relative to picture onset, but also in the duration of the visual stimulus. Thus, to correctly interpret behavioral data in Exp1M1right and Exp2M1left, we carried out Exp3Sham, which clarified that recognition accuracy in the two temporal conditions was comparable when no active stimulation of the right M1 was performed. These findings demonstrate a causal link between early right (but not left) M1 activity and visual perception. This link fits with the notion that sensorimotor networks in the right hemisphere support emotion and attention processing (Adolphs et al. 2000; Pourtois et al. 2004; Tamietto et al. 2006; Beraha et al. 2012) and appears also in line with the study of Pitcher et al. (2008) who found that TMS interference with early right somatosensory cortex activity (~100–170 ms) impaired visual recognition of facial expressions. While this latter study has been interpreted as strong evidence for embodied simulation accounts, it should be noted that the paradigm used by Pitcher et al. (2008) could not directly demonstrate the nature of somatosensory activation during emotion perception, because only behavioral data were acquired. In contrast, here we were able to show that when the right M1 appears critical for visual perception (~150 ms), no signs of motor resonance can be detected in that region, speaking against a major role of motor resonance processes—at least those that can be detected at 300 ms in bilateral M1—in the visual recognition of body expressions.

### Early orienting versus fight/flight motor reactions

While we found clear evidence for an early (~150 ms) orienting response in the right M1, in the same time window the left M1 showed a weak and marginally significant facilitation. This facilitation was specific for observation of fearful body expressions and did not correlate with accuracy in the emotion recognition task. In addition, the effect of left M1 stimulation on task performance did not differ from that of sham stimulation. While the non-significance of these findings needs to be interpreted with caution, the increase in left M1 excitability fits with previous work showing that watching threatening emotional scenes facilitates the excitability of the motor representation of the dominant hand in the left M1 (Borgomaneri et al. 2013), possibly reflecting the preparation of fast fight/flight motor reactions as hypothesized by evolutionary non-simulative accounts of emotion perception (Morris et al. 1999; Luo et al. 2007; Tamietto et al. 2009, 2012; de Gelder et al. 2010; LeDoux 2012). This suggests that, early in time, the right and left M1 may implement different neural processes supporting visual perception and adaptive fight/flight motor reactions, respectively.

It should be considered that TMS effects are site-specific but not site-limited (Fox et al. 1997; Ishikawa et al. 2007; Siebner et al. 2009; Avenanti et al. 2012a, b). Thus, it is possible that TMS modulated activity not only locally in M1, but also in other interconnected sensorimotor regions and that these regions contributed to the observed neurophysiological and behavioral effects. Nevertheless, our study suggests a dissociation between right and left sensorimotor networks in supporting perception of body expressions and implementing motor reactions to negative cues, respectively.

### Possible neural pathways supporting early orienting and perception of body expressions

We can only speculate about the neural networks supporting early motor orienting and visual perception of body expressions. On the one hand, right M1 may reflect the spillover of somatosensory activity associated with emotion perception (Winston et al. 2003; Leslie et al. 2004; Hennenlotter et al. 2005; Gazzola et al.^2012^) and the drop in accuracy found in Exp1M1right could be due to the spread of the TMS interference to closely interconnected right somatosensory regions (Fox et al. 1997; Ishikawa et al. 2007; Keysers et al. 2010) which in turn may have affected perception of emotional expressions (Adolphs 2002; Pourtois et al. 2004; Pitcher et al. 2008) and body movements (Jacquet and Avenanti 2013). More extensively, observation of emotional body expressions recruits a complex neural network which includes occipito-temporal and parieto-frontal somato-motor regions (de Gelder et al. 2004; Grèzes et al. 2007; Peelen et al. 2007; van de Riet et al. 2009; de Gelder et al. 2010; Tamietto and de Gelder 2010; Kret et al. 2011; Pichon et al. 2012), and these regions may provide a cortical pathway for fast orienting and visual perception of body expressions.

On the other hand, studies on brain damaged patients with permanent cortical blindness (Tamietto et al. 2009; Van den Stock et al. 2011) and imaging evidence from healthy individuals that subcortical structures (i.e., pulvinar, caudate nucleus and amygdala) are active during the perception of emotional bodies (van de Riet et al. 2009; de Gelder et al. 2010) suggest that the motor reaction to emotional bodies can be also implemented through subcortical routes (Tamietto and de Gelder 2010). These subcortical structures are anatomically connected with different segments of the motor pathway and may additionally provide signals to M1 during the processing of emotional bodies (Tamietto and de Gelder 2010; Tamietto et al. 2012). A possible role for subcortical networks in the perception of body expressions is also suggested by the evidence that TMS over visual cortex impairs visual recognition of neutral more than emotional body postures (Filmer and Monsell 2013), in line with the idea that emotion recognition can be supported by subcortical emotion-processing routes bypassing processing in the visual cortex (Morris et al. 1999; Liddell et al. 2005; Tamietto and de Gelder 2010; de Gelder et al. 2011). Thus, it is possible that disruption of the right M1 with TMS may have influenced subcortical activity critical for emotion processing, resulting in reduced orienting and impaired visual perception of body expressions, although it should be noted that our data suggest that TMS over right M1 interferes with perception of emotional and non-emotional bodies to a similar extent (Supplementary Table 1). Thus, if early orienting in the right M1 is mediated by subcortical networks, the causal involvement of such networks in visual recognition might be similar for emotional and non-emotional body expressions.

### Motor resonance with emotional and non-emotional body movements occurs later

As mentioned in the introduction, at 300 ms after stimulus onset the motor system is likely involved in action simulation: left M1 is modulated by action observation in the 250–350 ms range (Nishitani et al. 2004; Catmur et al. 2011; Barchiesi and Cattaneo 2013) and evidence indicates that motor resonance effects in M1 are mediated by those sectors of the premotor and parietal cortex that are recruited during action execution (Avenanti et al. 2007, 2013a, b; Koch et al. 2010; Catmur et al. 2011) and where mirror neurons were first recorded in the macaque brain (e.g., di Pellegrino et al. 1992; Gallese et al. 1996; Fogassi et al. 2005; Casile 2013). In a previous study using left M1 stimulation, we showed that MEPs recorded at 300 ms after stimulus onset increased in amplitude when a similar set of emotional and non-emotional body stimuli was presented (Borgomaneri et al. 2012). Confirming and extending this result, we found that, at 300 ms, seeing not only happy and fearful but also neutral body expressions increased the amplitude of MEPs relative to observing static neutral postures, and this was true not only for the left but also for the right M1, providing neurophysiological support for the notion that simulation-related activity is largely bilateral (Molnar-Szakacs et al. 2005; Keysers and Gazzola 2009; Caspers et al. 2010).

The motor facilitation detected during observation of neutral body movements did not significantly differ from that found with emotional bodies. Pictures of neutral gestures received lower emotional ratings than pictures of emotional body movements but, like the emotional expressions, were perceived as dynamic body postures. At 300 ms, motor excitability thus appears to be related to a simulation of the dynamic features of the observed expressions (Nishitani et al. 2004; Catmur et al. 2011; Barchiesi and Cattaneo 2013).

It should be noted, however, that fearful expressions received slightly lower implied motion ratings than happy or neutral movements, whereas motor facilitation was statistically comparable in the three dynamic conditions. This suggests that, at this stage, our measure of motor excitability is not sensitive to small differences in perceived implied motion and instead reflects a coarse categorization of the observed body posture as a dynamic or static body configuration.

Our study suggests that late markers of motor resonance in M1 do not play a major role in visual perception of body expressions. However, in this context, it is important to entertain the possibility that resonance processes necessary for perception could occur earlier in time (e.g., after 150 ms and before 300 ms) or in other anatomical locations (e.g., in premotor or parietal regions) without being immediately evident in M1 activity as measured by TMS (Jabbi and Keysers 2008; Avenanti and Urgesi 2011; Cattaneo et al. 2011; Tidoni et al. 2013; Urgesi et al. 2014). Thus, further studies are needed to test the time-course and causal involvement of motor resonance in visual perception of emotional body language.

### Influence of personality traits on orienting and motor resonance

The two sequential stages of motor system modulation were dissociated not only functionally and in time—with motor orienting being more involved in visual perception and occurring earlier—but also with respect to the influence that personality traits exert on these distinct stages of processing. The early right hemisphere reduction in motor excitability for emotional bodies was related to inter-individual differences in personal distress (PD) but not to the other IRI subscales (Davis 1996), which reflect more mature empathic dispositions. Personal distress is a self-focused aversive reaction to the negative state of another, and in line with our finding of a correlation with PD only at 150 ms, it is considered to be an early and rudimentary form of empathy, like emotional contagion (Davis 1996). Imaging studies have reported that participants who score high on the PD scale show enhanced reactivity of the insula when seeing happy and disgusted facial expressions (Jabbi et al. 2006) and when seeing painful expressions (Saarela et al. 2007), suggesting increased emotional reactivity to the emotions displayed by others. These findings are in line with ERP and imaging evidence that interpersonal anxiety-related dispositions are associated with a stronger visual cortex response to social and emotional information (Kolassa and Miltner 2006; Rossignol et al. 2012; Schulz et al. 2013). A relation between inter-individual differences in PD and increased neural reactivity at the motor level was also reported during observation of complex negative scenes (Borgomaneri et al. 2013) and others receiving painful stimulation (Avenanti et al. 2009a). Ferri et al. (2010) additionally showed that greater PD scores correlate with weaker motor control when facing emotional expressions. Taken together, these findings support the idea that interpersonal anxiety-related dispositions are associated with greater orienting and freezing-like responses to emotional cues, and that anxiety-related traits influence the way in which social and emotional signals are processed in the brain (Kret et al. 2011; Azevedo et al. 2013; Borgomaneri et al. 2013).

That the magnitude of early motor orienting in Exp1M1right correlated with both the TMS interferential effect and PD scores may raise the concern that unspecific factors (e.g., distractibility due to TMS that may in principle be greater in participants prone to experiencing personal distress) explain participants’ performance. However, PD scores did not correlate with the interferential effect and indeed these two variables were independent predictors of early motor orienting. This finding, together with the lack of interference with sham and left M1 stimulation, assures that unspecific effects cannot explain our results.

We additionally found that motor resonance in the two hemispheres marginally correlated with scores on a cognitive empathy subscale of the IRI, namely the Perspective Taking scale, which taps the ability to take the psychological perspectives of others. Although this finding was only marginally significant and should therefore be interpreted with caution, the correlation with PT is in line with (1) social psychology studies indicating that subjects who score high on the PT scale show high levels of automatic mimicry of postures, mannerisms and facial expressions during interpersonal communication (Chartrand and Bargh 1999); (2) the fMRI study of Gazzola et al. (2006) showing that activity in premotor and somatosensory regions responsive to both action perception and execution was predicted by inter-individual differences in PT scores; and (3) a series of other studies relating dispositional cognitive empathy to “resonant” activations (Pfeifer et al. 2008; Martínez-Jauand et al. 2012; Schaefer et al. 2012; Bolognini et al. 2013; see Bufalari and Ionta 2013 for a review). Neurophysiological studies have additionally shown that participants with high levels of PT and cognitive empathy show greater modulation of sensorimotor excitability during observation of actions (Lepage et al. 2010; Novembre et al. 2012) and pain (Cheng et al. 2008; Minio-Paluello et al. 2009; Avenanti et al. 2009a, 2010). Altogether, our findings and the above-mentioned results suggest that participants with greater cognitive empathy may show greater motor resonance during observation of others’ emotional expressions.

Thus, our study suggests that during observation of emotional body language, early motor orienting more than motor resonance supports visual recognition of body expressions. However, the finding of a relation between cognitive empathy and motor resonance may suggest that this later neural phenomenon—reflecting the embodiment of the observed body expression in the observers’ motor system—is involved in more sophisticated empathy-related understanding that occurs after visual recognition and may provide the observer with a reading of the body expressions ‘from the inside’ (Rizzolatti and Sinigaglia 2010).

## Conclusions

We provided correlational and causative evidence for a two-stage involvement of the motor cortex during perception of emotional body language. Our study suggests that, early in time, the right M1 actively supports perception of body expressions through the implementation of orienting responses, whereas the left M1 may be involved in preparing potential fight/flight motor reactions to negative cues. At a later stage, bilateral motor cortex activity reflects motor resonance mechanisms possibly linked to more sophisticated empathy-related processing.

## Electronic supplementary material

Below is the link to the electronic supplementary material.

Supplementary material 1 (DOCX 19 kb)
