# Supplementary material for: Transcranial magnetic stimulation reveals two functionally distinct stages of motor cortex involvement during perception of emotional body language
Source: Brain Struct Funct. 2014 Jul 15;220(5):2765–81. doi: 10.1007/s00429-014-0825-6 (PMC4549387; doi:10.1007/s00429-014-0825-6)
Supplement: Supplementary file 1 — Supplementary material 1 (DOCX 19 kb) [file 429_2014_825_MOESM1_ESM.docx]

**Transcranial magnetic stimulation reveals two functionally distinct stages of motor cortex involvement during perception of emotional body language.**

Borgomaneri S, Gazzola V & Avenanti A*

* Correspondence should be addressed to: Alessio Avenanti. University of Bologna and IRCCS Santa Lucia Foundation. E-mail: alessio.avenanti@unibo.it

**Supplementary Data**

Visual recognition of body expressions was compared in the three experiments. To this aim, accuracy scores were analyzed by means of a mixed model three-way ANOVA with Area (3 levels: Exp1M1right, Exp2M1left and Exp3Sham) as a between-subjects factor, and Time (2 levels: 150 and 300ms) and Movement type (4 levels: static, happy, neutral and fearful) as within-subjects factors. The ANOVA showed a main effect of Time (F(1,53) = 19.50, p < 0.0001) and Movement Type (F(3,159) = 19.22, p < 0.0001) and these main effects were qualified by a significant Time x Movement Type interaction (F(3,159) = 3.07, p = 0.03; see Supplementary Table 1). Importantly, the Time x Area interaction was also significant (F(2,53) = 3.57, p = 0.035). This was accounted for by the fact that in Exp1M1right, accuracy was lower in the early (150 ms) relative to the late (300 ms) temporal condition (92.8% vs 95.8%, *p* = 0.0006), whereas no similar change in performance was found in the other two experiments (*p* > 0.12). Notably, the Time x Area interaction was not qualified by the triple interaction (F(3,159) = 0.78, p = 0.59). Thus, accuracy was generally lower in the early relative to the late conditions of Exp1M1right and this effect was similar across visual conditions (Supplementary Table 1).

**Supplementary Table 1**

|  | |  | **150 ms** | |  |  | **300 ms** | | | |  | |
| --- | --- | --- | --- | --- | --- | --- | --- | --- | --- | --- | --- | --- |
|  | |  |  | |  |  |  | |  | | | |
|  | Static | Happy | Neutral | Fearful | Static | | Happy | | Neutral | | Fearful | |
|  |  |  |  |  |  | |  | |  | |  | |
| Exp1M1right | 99.0 ± 2.4 | 91.7 ± 12.2 | 87.0 ± 10.9 | 93.7 ± 6.7 | 98.7 ± 2.7 | | 93.7 ± 9.3 | | 93.3 ± 6.8 | | 97.3 ± 4.0 | |
|  |  |  |  |  |  | |  | |  | |  | |
| Exp2M1left | 99.7 ± 1.5 | 94.0 ± 12.0 | 86.3 ± 10.7 | 96.3 ± 5.9 | 100.0 ± 0.0 | | 93.7 ± 10.0 | | 88.0 ± 9.6 | | 97.0 ± 4.0 | |
|  |  |  |  |  |  | |  | |  | |  | |
| Exp3Sham | 98.8 ± 3.6 | 90.8 ± 6.8 | 91.7 ± 7.5 | 95.0 ± 5.7 | 98.8 ± 2.7 | | 91.7 ± 8.6 | | 93.8 ± 7.9 | | 97.9 ± 5.3 | |

**Supplementary Table 1** Mean ± standard deviation of accuracy scores (% correct responses) in the recognition task during observation of static, happy, neutral and fearful body postures in the first experiment (Exp1M1right), the second experiment (Exp2M1left) and the third control experiment (Exp3Sham) with TMS delivered at 150 or 300 ms after stimulus onset
